# Robust analysis of stepped wedge trials using cluster‐level summaries within periods

**DOI:** 10.1002/sim.7668

**Published:** 2018-04-10

**Authors:** J.A. Thompson, C. Davey, K. Fielding, J.R. Hargreaves, R.J. Hayes

**Affiliations:** ^1^ Department of Infectious Disease Epidemiology London School of Hygiene and Tropical Medicine London UK; ^2^ MRC London Hub for Trials Methodology Research London UK; ^3^ Department of Public Health, Environments and Society London School of Hygiene and Tropical Medicine London UK

**Keywords:** cluster randomised trial, confidence interval coverage, permutation test, simulation study, stepped wedge trial

## Abstract

In stepped‐wedge trials (SWTs), the intervention is rolled out in a random order over more than 1 time‐period. SWTs are often analysed using mixed‐effects models that require strong assumptions and may be inappropriate when the number of clusters is small.

We propose a non‐parametric within‐period method to analyse SWTs. This method estimates the intervention effect by comparing intervention and control conditions in a given period using cluster‐level data corresponding to exposure. The within‐period intervention effects are combined with an inverse‐variance‐weighted average, and permutation tests are used. We present an example and, using simulated data, compared the method to (1) a parametric cluster‐level within‐period method, (2) the most commonly used mixed‐effects model, and (3) a more flexible mixed‐effects model. We simulated scenarios where period effects were common to all clusters, and when they varied according to a distribution informed by routinely collected health data.

The non‐parametric within‐period method provided unbiased intervention effect estimates with correct confidence‐interval coverage for all scenarios. The parametric within‐period method produced confidence intervals with low coverage for most scenarios. The mixed‐effects models' confidence intervals had low coverage when period effects varied between clusters but had greater power than the non‐parametric within‐period method when period effects were common to all clusters.

The non‐parametric within‐period method is a robust method for analysing SWT. The method could be used by trial statisticians who want to emphasise that the SWT is a randomised trial, in the common position of being uncertain about whether data will meet the assumptions necessary for mixed‐effect models.

## 1 BACKGROUND

Parallel cluster‐randomised trials (CRTs) with sufficient numbers of clusters can be analysed in 2 ways: at the cluster level using cluster‐level summaries of observation‐level data, or by accounting for correlation in the observation‐level data using statistical models. Cluster‐level methods are robust and allow simple computation of risk differences and risk ratios, which are easier to interpret than odds ratios unless outcomes are rare.^1^, ^2^ By contrast, observation‐level models with binary outcomes are less robust to small numbers of clusters.^3^

Stepped‐wedge trials (SWTs) randomise clusters to sequences.^4^ Each sequence introduces the intervention at different times, so that clusters allocated to each sequence are in the control condition for a unique length of time before switching to the intervention condition for the remainder of the study. The times between when the clusters in 1 sequence switch and clusters in the next sequence switch are referred to as “periods”. Outcome data are typically collected for all periods 5 and sometimes also before the clusters in the first sequence starts receiving the intervention, and after the last sequence starts to receive the intervention. Outcomes are assigned to the period in which the individual was exposed. These may occur within the period but may occur during a later period 5 or even after the rollout is complete.^6^

The intervention effect can be estimated from 2 “directions” of comparison: within periods or between periods.

Within‐period comparisons are akin to a parallel CRT because each cluster is in 1 condition only, determined at random. For each period, observations corresponding to exposure to the control condition are compared with observations corresponding to exposure to the intervention condition. These are often known as “vertical” comparisons.

Between‐period comparisons compare observations corresponding to control and intervention conditions within each cluster across periods. Between‐period comparisons are confounded with changes in participation and in the outcome over time (secular trends or “period effects”) because each cluster is in the intervention condition later than the control condition.^5^ Analyses that incorporate between‐period comparisons must adjust for period effects and must also make assumptions about the correlation of observations within each cluster. These are also known as horizontal comparisons.

SWTs are most commonly analysed using mixed‐effect models that combine within‐period and between‐period comparisons.^7^ These have been shown to be sensitive to misspecification of the random effects 8, 9, 10: they can produce biased estimates and underestimate standard errors. The correlation structure of the data is usually not known in advance of a trial. To ensure a valid analysis is pre‐specified, it may be safer to plan an analysis that does not require these assumptions.

Permutation tests are 1 way to account for correlations without specifying the correlation structure within clusters; Ji et al^9^ and Wang and DeGruttola 10 suggest using a mixed‐effect model for effect estimation and permutation tests for calculating confidence intervals (CIs) and *P*‐values. Although they find this method to be more robust than a mixed‐effect model alone, this requires that the mixed‐effect model provides an unbiased estimate of the treatment effect and this is not always the case.^8^

An alternative approach to estimating an intervention effect is to combine within‐period comparisons.^7^ Published literature has suggested combining within‐period comparisons using inverse variance weights.^4^, ^11^, ^12^ We hypothesise that such a method will be a more robust way to estimate an intervention effect because this does not require assumptions about, or estimation of, a correlation structure. Formulae for estimating a parametric variance for this combined estimate are also given in this literature. These either require assuming a correlation structure,^11^ or estimating this structure from the data 4, 12; these can be problematic if the assumed structure is incorrect, or if there are insufficient clusters per sequence to estimate the correlations.

In this paper, we introduce a cluster‐level, within‐period, analysis method that uses permutation tests. We contrast this against a parametric cluster‐level within‐period approach,^12^, ^13^ the most commonly used mixed‐effects model,^14^ and a more flexible mixed‐effects model. As illustration, we applied the methods to data from a SWT assessing the effect of a new tuberculosis (TB) diagnostic test on patient outcomes. Using simulations based on routinely collected data, we assessed the performance of the methods in terms of bias, coverage, and power.

## 2 ANALYSIS METHODS

We compared 4 methods for analysing SWTs. Two use only the within‐period comparisons and analyse at the cluster‐level (referred to as within‐period methods), and 2 are mixed‐effect models that incorporate the between‐period comparisons and analyse at the observation‐level (referred to as mixed‐effect models). We focused on a binary outcome, which is common in SWTs.^7^

### 2.1 Within‐period methods

Because within‐period comparisons are not possible during periods where all clusters are in 1 condition, these methods only use data collected after clusters allocated to the sequence with the earliest introduction of the intervention switch to the intervention and before clusters allocated to the sequence with the latest introduction of the intervention switch to the intervention. In contrast to currently published within‐period analyses,^11^ these methods do not make assumptions about the correlation of observations between periods.

#### 2.1.1 Non‐parametric within‐period method

For this method, we use a within‐period cluster‐level analysis, with permutation tests to calculate CIs and *P*‐values.

The method uses the following 3 stages (see R code in Supporting Information A):

1. First, we calculate a summary of the outcome, *Y*_*ij*_, in each cluster *i* corresponding to exposure to the control or intervention conditions during period *j*; for binary outcomes, these could be log odds (to calculate odds ratios), log risks (to calculate risk ratios), or risks (to calculate risk differences). Other summary measures can be used for different outcome types.
2. Next, we calculate the period‐specific intervention effect, ${\hat{\theta }}_{j}$, as the difference between the mean cluster‐period summaries for the intervention and the control conditions during period *j*:
  $${\hat{\theta }}_{j}=\frac{1}{c_{1j}}\sum_{i:X_{\mathrm{ij}}=1}Y_{\mathrm{ij}}-\frac{1}{c_{0j}}\sum_{i:X_{\mathrm{ij}}=0}Y_{\mathrm{ij}}$$
  where *c*_1*j*_ and *c*_0*j*_ are the numbers of clusters in the intervention condition and the control condition, respectively, during period *j*, and *X*_*ij*_ is an indicator denoting whether cluster *i* was allocated to receive the intervention or control condition during period *j*, equal to 1 for intervention and 0 for control.
3. Last, we combine the period‐specific intervention effect estimates using an inverse‐variance weighted average, to give an overall estimated intervention effect $\hat{\theta }$:
  $$\hat{\theta }=\sum_{j=1}^{j=J}\frac{w_{j}}{w}{\hat{\theta }}_{j}$$
  where $w=\sum_{j=1}^{j=J}w_{j}$, using weights based on the pooled variance of the period‐specific estimated intervention effect allowing for the unequal number of clusters in each condition. These weights are calculated as:
  $$w_{j}=\hat{\mathrm{Var}}{\left({\hat{\theta }}_{j}\right)}^{-1}={\left[\left(\frac{\left(c_{0j}-1\right)s_{0j}^{2}+\left(c_{1j}-1\right)s_{1j}^{2}}{c_{0j}+c_{1j}-2}\right)\left(\frac{1}{c_{0j}}+\frac{1}{c_{1j}}\right)\right]}^{-1}$$
  where $s_{0j}^{2}$ and $s_{1j}^{2}$ are the empirical variances of the cluster‐period summaries in the control and intervention conditions, respectively, for each period *j*.

We use a permutation test to calculate *P*‐values and CIs, which requires no assumptions about the correlation between the effects in each period. We randomly permute the assignment of clusters to sequences and therefore the time at which clusters switch from control to intervention conditions. We calculate an intervention effect for each permutation, as described previously. The *P*‐value for the null hypothesis of no intervention effect is given by the proportion of permutations with an estimated intervention effect the same as or more extreme than that observed. We use 1000 permutations to allow us to calculate *P*‐values to 3 decimal places.

Permutation tests can be used to generate 95% CIs using an iterative process. The following process is conducted iteratively for several given intervention effect values to find the values that return a 1‐sided *P*‐value of 0.025: the upper and lower confidence limits.

First, we test for evidence against a given intervention effect *θ*_*A*_ by subtracting it from the observed cluster‐period summaries in the intervention condition:

$${Y_{\mathrm{ij}}}^{*}=\left\{\begin{array}{c} Y_{\mathrm{ij}}-\theta_{A}\;\text{if}\;i:X_{\mathrm{ij}}=1\\ Y_{\mathrm{ij}}\;\text{if}\;i:X_{\mathrm{ij}}=0. \end{array}\right.$$

Then, using the new intervention‐condition cluster‐period summaries and the original control‐condition cluster‐period summaries, $Y_{\mathrm{ij}}^{*}$, we estimate an intervention effect as described earlier and permute the assignment of clusters to sequences to generate the *P*‐value.

The method assumes that the clusters are independent of one another within each period and assumes exchangeability of observations within clusters within each period. No assumptions are made about correlations between observations or cluster summaries over different periods, and no assumptions are made about the period effects. We assume that the intervention effect is the same for all clusters in all periods.

Outcomes of different types can be analysed, and several different intervention effects can be estimated. In this paper, we focus on calculating odds ratios to make comparisons with logistic mixed‐effect models. This means we calculate log odds of the cluster summaries:

$$Y_{\mathrm{ij}}=\log\left(\frac{p_{\mathrm{ij}}}{1-p_{\mathrm{ij}}}\right)$$

where *p*_*ij*_ is the proportion of individuals in cluster *i* during period *j* with the outcome of interest. The log odds is not defined for clusters with a proportion of 1 or 0. To include these clusters, we used a heuristic adjustment, adding 0.5 to both the number of individuals with the outcome of interest and the number of individuals without the outcome, in the affected clusters only.^15^ We will also demonstrate calculation of a risk difference in the example; the risk difference does not require the heuristic adjustments.

#### 2.1.2 Parametric within‐period method

Several papers have described a method of combining period‐specific intervention effects in the context of longitudinal data.^12^, ^13^, ^16^ This method could also be used for the analysis of SWTs. We applied this method using a cluster‐level analysis for comparability with the non‐parametric within‐period method. The method uses the following 4 stages:

1. Cluster‐period summaries are calculated as in the non‐parametric within‐period method, earlier, with the use of heuristic adjustments when necessary.
2. Generalised estimating equations, assuming a normal distribution of the cluster summaries, robust standard errors, and an independent working correlation matrix on data from across the trial periods are used to calculate period‐specific intervention effects, using the following model:
  $$Y_{\mathrm{ij}}=\mu_{j}+\theta_{j}X_{\mathrm{ij}}+\epsilon_{\mathrm{ij}}$$
  where *Y*_*ij*_ are summaries of the outcome in each cluster‐period, *μ*_*j*_ is the mean of the cluster‐period summaries in period *j*, *θ*_*j*_ is the intervention effect in period *j*, and *ϵ*_*ij*_ are the residuals.
3. The resulting period‐specific intervention effects, ${\hat{\theta }}_{j}$, are combined as
  $$\hat{\theta }=\sum_{j=1}^{j=J}w_{j}{\hat{\theta }}_{j}$$
  where $w=\left[w_{1},\ldots ,w_{J}\right]={\left(\left[1,\ldots ,1\right]{\hat{\Lambda }}_{w}^{-1}\left[1,\ldots ,1\right]\right)}^{-1}{\hat{\Lambda }}_{w}^{-1}\left[1,\ldots ,1\right]$, and ${\hat{\Lambda }}_{w}$ is the covariance matrix for the period‐specific parameters ${\hat{\theta }}_{j}$ estimated by the generalised estimating equations.
4. The resulting intervention effect estimate follows a normal distribution with variance $\hat{w}\hat{\Lambda }\hat{w}$ and so confidence intervals and *P*‐values can be constructed parametrically.

As with the non‐parametric within‐period method, we focus on estimation of an odds ratio, *Y*_*ij*_ =  *log* (*p*_*ij*_/(1 − *p*_*ij*_)), but other intervention effects can be calculated, and other outcome types can be analysed in a similar way.

### 2.2 Mixed‐effects models

We used 2 mixed‐effect models that have been suggested for SWT analysis. We used the normal distribution for the calculation of CIs.

#### 2.2.1 Cluster mixed‐effect model

The first model was described by Hussey and Hughes.^17^ This model has been shown to produce CIs that are too narrow when misspecified^8^, ^9^, ^10^ and so is now thought to be unsuitable for general use in the analysis of SWTs. It is included here because it is commonly used in practice. It has been adapted to a logistic model for a binary outcome as:

$$\text{logit}\left(P\left(Y_{\mathrm{ijk}}=1\right)\right)=\mu +\beta_{j}+\theta X_{\mathrm{ij}}+u_{i}$$

where *Y*_*ijk*_ is the outcome in individual *k* at time *j* in cluster *i*, *μ* is the log‐odds of the outcome in the first period in the control condition, *β*_*j*_ is the change in the outcome from the first period to period *j*, *θ* is the overall intervention effect, and $u_{i}∼N\left(0,\sigma_{u}^{2}\right)$ is a random effect for cluster *i*.

This model assumes that clusters are independent of one another; that the intervention effect and period effects are common to all clusters; and that observations are equally correlated within clusters across all periods.

#### 2.2.2 Cluster‐period mixed‐effect model

A more flexible mixed‐effects model has been recommended for use with SWTs 18, 19, 20:

$$\text{logit}\left(P\left(Y_{\mathrm{ijk}}=1\right)\right)=\mu +\beta_{j}+\theta X_{\mathrm{ij}}+u_{i}+q_{\mathrm{ij}.}$$

where $u_{i}∼N\left(0,\sigma_{u}^{2}\right)$ is a random effect for each cluster, and $q_{\mathrm{ij}}∼N\left(0,\sigma_{q}^{2}\right)$ is a random effect for each cluster‐period. Using this model, observations in the same cluster and same period are more correlated than observations in the same cluster but different periods. However, the correlation of observations in different periods is the same for each pair of periods, and the total variance is fixed to be the same in all periods.

There is a growing literature on designing SWTs that use this model,^18^, ^19^ but there is currently no assessment of its use in trial analysis.

## 3 APPLICATION TO TB DIAGNOSTIC TRIAL

We applied the 4 methods described previously to a SWT conducted in Brazil that assessed the effect of a new TB diagnostic test on outcomes of adults started on TB treatment.^21^

### 3.1 Description of the study

The new diagnostic test (Xpert MTB/RIF) is more sensitive than the standard smear microscopy method and provides a result for rifampicin drug resistance.^22^ Clusters were 14 laboratories used to diagnose TB in 2 cities in Brazil. Patients who were diagnosed with TB in these laboratories during an 8‐month period were enrolled, and their treatment outcome followed up for between 15 and 23 months after diagnosis. Unfavourable patient outcomes were defined as death, lost‐to‐follow‐up during treatment, transfer out of clinic (including to more specialist centres for those not responding to treatment), or suspected drug resistance. In the first month, all the laboratories were in the control condition using sputum smear microscopy. At the start of each subsequence month, pairs of laboratories started using the new diagnostic technology in a random order. In the final month, all laboratories were using the new diagnostic test. Unlike the published analysis of this trial, we do not include data from the first and final months, where all laboratories were in the control condition, or where they were all in the intervention condition, respectively. We estimated, using the cluster mixed‐effect model within each period, that patients' outcomes had an intra‐cluster correlation coefficient (ICC) of 0.003 throughout the trial.

### 3.2 Results

In the published report of the TB diagnostic trial,^21^ the authors found no evidence that the new diagnostic test reduced unfavourable outcomes among 3926 TB patients. They used a mixed‐effects model with a fixed effect for the intervention, and a random effect for cluster (crude OR = 0.92 95% CI 0.79, 1.06; a similar result was found in analysis adjusting for sex, age, city, HIV status, and diagnosis status). Neither analysis adjusted for period effects. We found a similar result when we repeated this analysis on our subset of 3009 TB patients that excludes data from the period where all laboratories were in the control or all were in the intervention condition (results not shown).

All methods gave similar estimates of the odds ratio (see upper panel of Table 1), but the strength of evidence against no effect differed: from *P* = 0.02 (both within‐period methods) to *P* = 0.10 (both mixed‐effect models). We used the non‐parametric and parametric within‐period methods to estimate the risk difference (see lower panel of Table 1), which gave similar estimates of effect (risk differences of −4.8% and − 4.2%, respectively); however, the evidence against no effect was different, with *P* = 0.04 for the non‐parametric method and *P* = 0.007 for the parametric method.

**Table 1:** The impact of introducing a new TB diagnostic test to laboratories on unfavourable outcomes among TB patients, using different analysis methods. Mixed‐effects models used observation‐level data; within‐period methods used cluster‐level data

Analysis Method	Intervention Effect	*P*‐Value
	Odds ratio	
Non‐parametric within‐period method	0.78 (0.61, 0.96)	0.02
Parametric within‐period method	0.85 (0.74, 0.98)	0.02
Cluster mixed‐effect model	0.83 (0.67, 1.03)	0.10
Cluster‐period mixed effect model	0.83 (0.67, 1.03)	0.10
	Risk difference	
Non‐parametric within‐period method	−4.8% (−10.0%, −0.3%)	0.04
Parametric within‐period method	−4.2% (−7.2%, −1.1%)	0.007

Table 2 shows the 3 stages for calculating the risk difference using the non‐parametric within‐period method. The risk difference for the intervention effect ranged from +1.7% to −10.0% across the 6 periods, and the overall estimated risk difference, combining estimates across the 6 periods with inverse variance weights, was −4.8%; that is a 4.8% reduction in the risk of an unfavourable outcome using the new diagnostic test. Permutation tests gave a 95% CI of (−10.0%, −0.3%) with some evidence of an effect (*P* = 0.04).

**Table 2:** The estimated risk difference and its components, for each step using the non‐parametric within‐cluster method. The mean and variance of cluster level risks were calculated for each condition in each period, which were then used to calculate the risk difference and its variance in each period, which were summarised with an inverse‐variance weighted average

Stage		Period					
		1	2	3	4	5	6
(1)	Control (smear microscopy) arm:						
	Number of clusters	12	10	8	6	4	2
	Mean of cluster‐level risks	30.5%	26.5%	33.9%	32.4%	40.7%	38.4%
	Intervention (Xpert MTB/RIF) arm:						
	Number of clusters	2	4	6	8	10	12
	Mean of cluster‐level risks	32.2%	23.8%	23.9%	30.2%	33.3%	34.6%
(2)	Risk difference	+1.7%	−2.7%	−10.0%	−2.2%	−7.4%	−3.7%
(3)	Control arm: Variance of cluster‐level risks	0.009	0.005	0.006	0.005	0.012	0.036
	Intervention arm: Variance of cluster‐level risks	0.040	0.015	0.001	0.002	0.007	0.020
	Relative weight (w_j_/ ∑ w_j_)	0.05	0.13	0.27	0.41	0.11	0.03
	Weighted average of risk differences	−4.8%					

## 4 SIMULATION STUDY

We performed a simulation study to investigate the performance of our 4 methods in scenarios with different period effects and ICCs, and for several SWT designs.

### 4.1 Data

**Figure 1:**
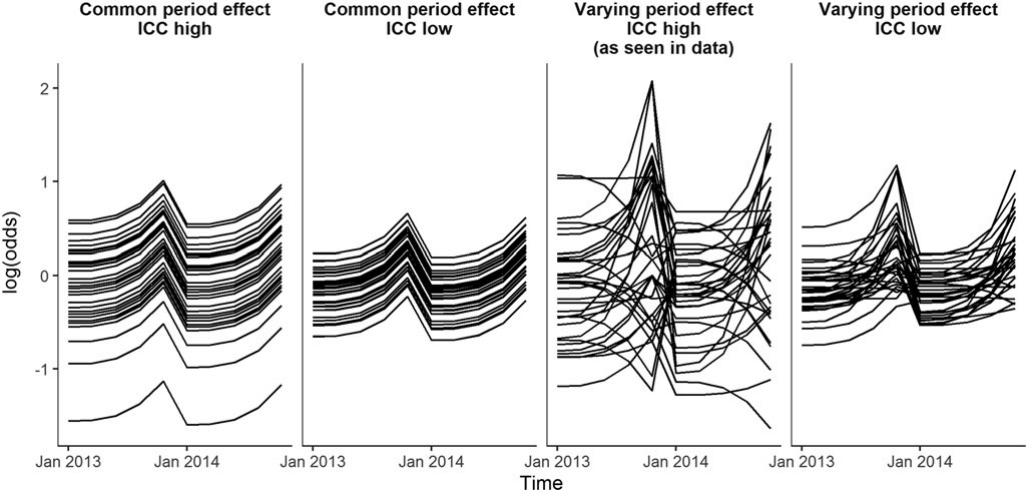
Simulated log‐odds for 33 clusters over 2 years with no intervention in the 4 scenarios; common period effects with a high ICC (0.08), common period effects with a low ICC (0.02), varying period effects with a high ICC (0.08), and varying period effects with a low ICC (0.02)

We used local‐authority‐level data on uptake of National Health Service health checks in England in 2013 to 2014 to inform the simulation study. Health checks were offered to all adults aged 40 to 74 every 5 years by GPs and third parties to assess risk of diabetes, heart disease, kidney disease, stroke, and dementia.^23^ Details of how we generated the scenarios to be used in the simulation study from these data are given in Supporting Information B. The mean of the local authority‐level percentage of patients accepting health checks when offered was 49% in the first quarter of 2013; this increased to 54% in the last quarter. At the start of 2014, the mean was 46%; this increased to 56% in the last quarter. We used these health check data to generate 4 scenarios (Figure 1).

We generated period effects that were common to all clusters, and period effects that varied between clusters to the degree that was observed in the data. We expected the within‐period methods to be unbiased and have correct coverage in both the common and the varying period effects scenarios.

We assessed the power of the methods using 2 scenarios with different values of ICC. For one, we used the between‐cluster variability observed in the data (*ICC* ≈ 0.08 in the first quarter of 2013, hereafter referred to as “high ICC”), and for another we used one fifth of the observed between‐cluster variability (*ICC* ≈ 0.02 in the first quarter of 2013, hereafter referred to as “low ICC”).

When the period effects varied between the clusters, the between‐cluster variance changed over time. Therefore, the ICC changed over time. Over the 2 years, the ICC varied between 0.06 and 0.19 for the high ICC and varying period effects scenario, as observed in the data, and between 0.01 and 0.04 for the low ICC and varying period effects scenario.

### 4.2 Trial designs

In each of the 4 scenarios (common and varying period effect and high and low ICC), we simulated SWTs of an intervention designed to increase acceptance of health checks when offered by a GP. The intervention effect had an odds ratio of 1.3 (log‐odds ratio = 0.26), favouring the intervention.

**Figure 2:**
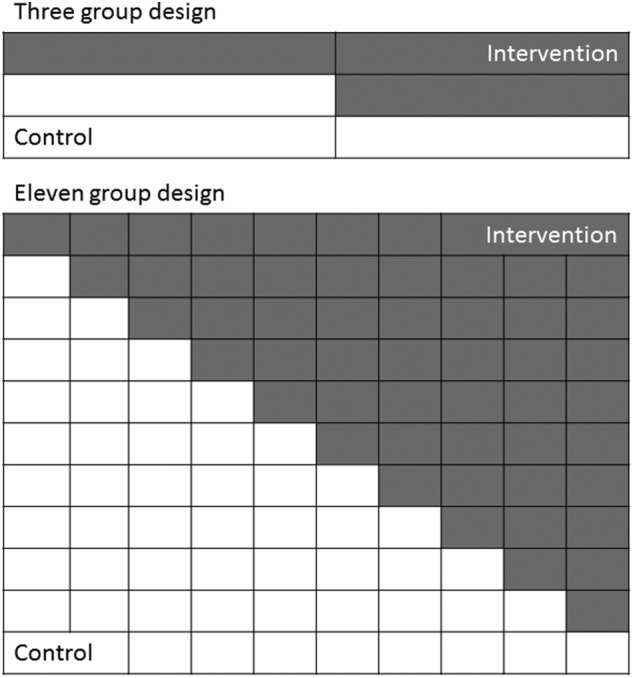
Diagrams of the 3 and 11 sequence designs. For each, we simulated trials with 3 or 11 clusters per sequence

We applied 4 trial designs to each of the 4 scenarios to assess how the numbers of sequences, the number of clusters per sequence, and the total number of clusters affected the performance of the methods. The 4 trial designs had either 3 or 11 sequences with either 3 or 11 clusters per sequence (Figure 2). This resulted in trial designs with a total of 9 clusters (3 sequences with 3 clusters per sequence), 33 clusters (3 sequences with 11 clusters per sequence, or 11 sequences with 3 clusters per sequence), or 121 clusters (11 sequences with 11 clusters per sequence). Unlike the mixed‐effect models, the within‐period methods require clusters in both the control and intervention condition in each period, and therefore we simulated designs that started when clusters in the first sequence switched to the intervention and stopped when clusters in the final sequences switched to the intervention (Figure 2).

The total number of observations for each cluster across the trial was selected from a log‐normal distribution (*μ*=5.3, *σ*^2^=0.25) regardless of the trial design; this gave a median total cluster size of 200 (interquartile range 143‐281) with observations evenly distributed across the periods. This gave a median total sample size of 1800 for designs with 9 clusters, 6600 for designs with 33 clusters, and 24 200 for designs with 121 clusters. In scenarios with common period effects, the smallest trial (9 clusters) had 35% power to detect an odds ratio of 1.3 with the cluster mixed‐effects model, and the largest trial (121 clusters) had >99.9% power.^24^

Each of the 4 trial designs for each of the 4 scenarios was simulated 1000 times, allowing us to estimate coverage of 95% CIs to within 1.4%.

### 4.3 Evaluation of methods

We analysed each simulated trial using both within‐period methods and both the mixed‐effects models. We compared the 4 methods in terms of bias, coverage, and power for each trial design and scenario, in line with recommendations from Burton et al.^25^

We calculated the proportion of within‐period analyses that required the heuristic adjustment and the proportion of mixed‐effects analyses that converged. Bias was calculated as the deviation of the mean of the estimates from the true log‐odds ratio. Effect estimates within half a standard deviation of the true effect were considered unbiased.^25^ We compared the variability of the estimates given by each method using the ratio of the variances. The coverage of the 95% CIs was calculated as the proportion of simulations with *P* > 0.05 against the true effect, ie, the proportion of CIs that contained the true effect. We calculated the power to detect an effect at 5% significance as the proportion of simulations with *P* < 0.05 against no intervention effect.

### 4.4 Simulation study results

For all simulations, the within‐period methods provided effect estimates and *P*‐values, all of the cluster mixed‐effect models converged, and all but 2 of the cluster‐period mixed‐effects models converged. For the cluster‐level analyses, the heuristic adjustment was used in 84% to 100% of simulations with a trial design of 11 clusters per sequence and 11 sequences (mean of 2‐7 times per simulation), 10% to 64% with 3 clusters per sequence and 11 sequences (mean of 0‐1 times per simulation), once with 11 clusters per sequence and 3 sequences, and was not used with 3 clusters per sequence and 3 sequences. See Supporting Information C for more details.

#### 4.4.1 Bias and variability

**Figure 3:**
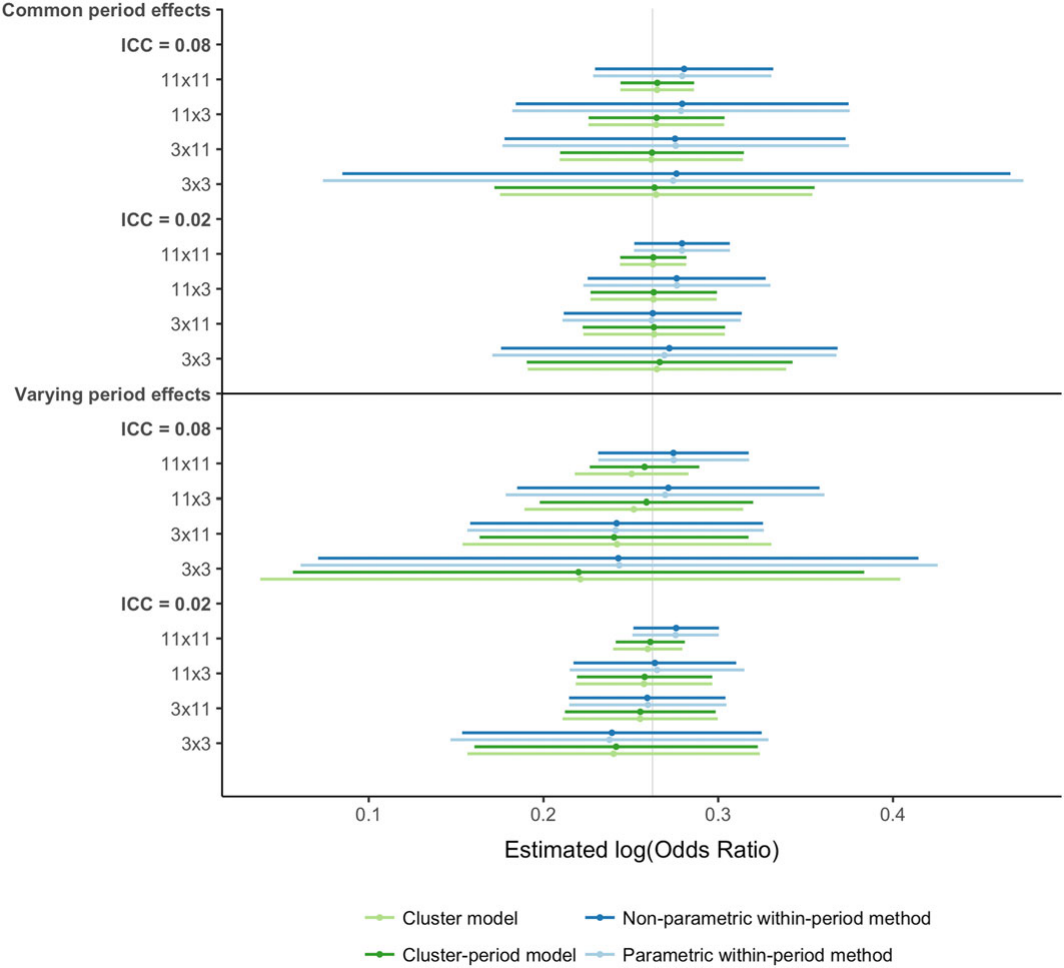
Graph of mean and ½ standard deviation either side of the estimated log‐odds ratios for each method, scenario, and trial design. The vertical grey line denotes the true log‐odds ratio. The mean estimates are shown with the circles. The horizontal lines depict ½ standard deviation either side of the mean. The effects are organised first by whether the period effects vary between cluster, then by the ICC, and finally by the trial design (number of sequences x the number of clusters allocated to each sequence) [Colour figure can be viewed at http://wileyonlinelibrary.com]

Figure 3 shows the mean and half a standard deviation either side of the mean of the estimated intervention effects for each analysis method, scenario, and trial design. All analysis methods gave unbiased intervention effects in all scenarios and trial designs (see Supporting Information D).

The mixed‐effect models' estimates had similar variability, and the within‐period methods' estimates also had similar variability. The within‐period methods' estimates were between 1.56 and 6.18 times more variable than the mixed‐effects models' estimates when period effects were common to all clusters. When period effects differed between clusters, the variability of the estimates was more alike between analyses: the within‐period methods' estimates were between 0.88 and 2.23 times as variable as the mixed‐effect models' estimates. This difference between the common‐period‐effect scenarios and varying‐period‐effect scenarios was largely driven by an increase in variability of mixed‐effects models' estimates. In all scenarios, the difference in variability was greater when there were more sequences because of a reduction in variability in the mixed‐effect models estimates, and when the ICC was high because of an increase in variability in the within‐period methods' estimates.

#### 4.4.2 Coverage

**Figure 4:**
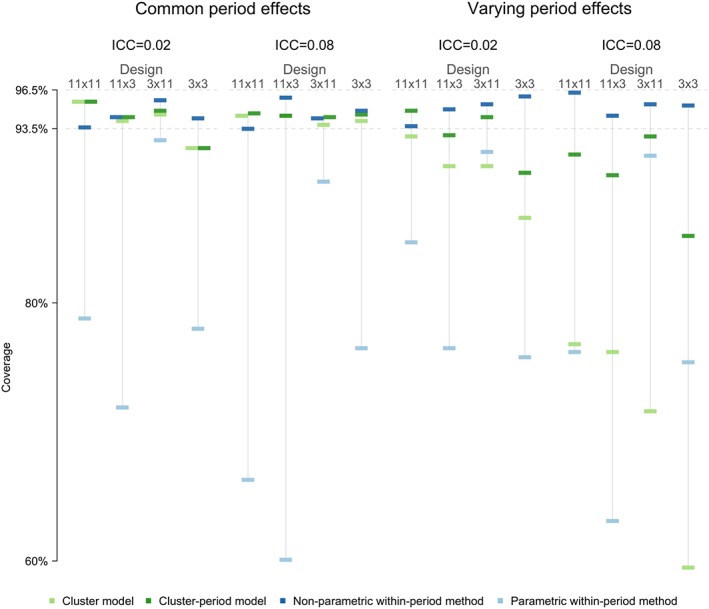
Coverage of 95% confidence intervals for each analysis, scenario, and trial design. Coverage between 96.4% and 94.6% is nominal. The coverage estimates are organised first by whether the time‐trends vary between cluster, then by the ICC, and finally by the trial design (number of sequences x number of cluster allocated to each sequence) [Colour figure can be viewed at http://wileyonlinelibrary.com]

Figure 4 shows the coverage of the 95% CIs for each method, scenario, and trial design (see Supporting Information E for further details).

The non‐parametric within‐period method performed well; it achieved 94% to 96% coverage for both common and varying period effects, both levels of ICC, and all trial designs.

The parametric within‐period method performed badly; it had poor 95% CI coverage in all scenarios considered, particularly for designs with 3 clusters per sequence. For the mixed‐effects models, both gave reasonable coverage (92%‐96%) when period effects were common to all clusters (shown in the left half of Figure 4), although they may have 95% CIs with low coverage in scenarios with 9 clusters and low ICC (92% coverage). However, when period effects varied between clusters (shown in the right half of Figure 4), both produced under‐coverage of 95% CIs. The degree of under‐coverage was severe for the cluster mixed‐effect model, particularly when the ICC was high (60%‐77% coverage and 87%‐93% coverage for high and low ICC, respectively). For designs containing 33 or more clusters, the cluster‐period mixed‐effect model achieved nominal coverage with a low ICC (93%‐95%) but gave low coverage with a high ICC (90%‐93%). For designs with fewer than 33 clusters, the cluster‐period model produced under‐coverage of the 95% CI (85%‐90%).

#### 4.4.3 Power

Table 3 shows the power for each method, scenario, and trial design (see Supporting Information F for further details).

**Table 3:** Power for each method, scenario, and trial design

Period Effects	ICC	Sequences	Clusters per Sequence	Power			
				Non‐parametric within‐period method	Parametric within‐period method	Cluster model	Cluster‐period model
Common	0.02	3	3	26.9	51.7	48.7	47.1
	0.02	3	11	72.8	77.4	91.8	90.1
	0.02	11	3	78.1	93.9	96.4	96.4
	0.02	11	11	99.8	100	100	100
	0.08	3	3	11.3	32.9	35.5	33.9
	0.08	3	11	29.6	35.4	73.1	70.5
	0.08	11	3	32.3	74.1	93.4	93.4
	0.08	11	11	79.5	95.4	100	100
Varying	0.02	3	3	22.4	48.7	45.0	39.9
	0.02	3	11	79.5	85.3	89.0	85.2
	0.02	11	3	79.9	93.0	95.4	94.5
	0.02	11	11	100	100	100	100
	0.08	3	3	10.5	32.4	45.3	22.1
	0.08	3	11	27.9	35.5	63.2	36.3
	0.08	11	3	35.7	73.0	80.4	67.9
	0.08	11	11	87.0	98.0	99.8	99.2

The power of the non‐parametric within‐period method ranged from 10% to 100%. Power was higher with more clusters and lower ICC. For example, with common period effects, 11 sequences, and 3 clusters per sequence, the method had 32% and 78% power for high and low ICC, respectively. Whether period effects were common or varying had limited impact on the power.

The power of the parametric within‐period method will not be discussed because of its poor 95% CI coverage for all scenarios.

When period effects were common, the non‐parametric within‐period method had lower power than both mixed‐effects models. The difference ranged from 0% to 61% less power; power loss was greatest when ICC was high and with 11 sequences. The 2 mixed‐effect models had similar power.

When period effects were varying, the under‐coverage of the CIs described above means that the power for the mixed‐effects models will be overestimated. However, for the sake of making some comparisons, we consider the cluster‐period model, because it had closer to nominal coverage than the cluster mixed‐effects model. The difference in power between the cluster‐period model and the non‐parametric within‐period when the period effects were common ranged from 2% to 41.8% difference; when the period effects were varying, the differences ranged from 0% to 37.3%. The larger of those differences were when the ICC was high, which was also when the cluster‐period model had lowest coverage and therefore power would be artificially inflated.

## 5 DISCUSSION

We have presented a novel non‐parametric within‐period method to analyse SWTs and compared this with a parametric within‐period analysis method, a commonly used mixed‐effects model, and a more flexible mixed‐effects model. Of the 4 methods considered, the non‐parametric within‐period method was the only approach to consistently provide correct CI coverage, although all of the methods gave unbiased point estimates. The lack of nominal coverage from the other methods in some or all of the scenarios considered made most comparisons of the power of the methods invalid. Where comparisons were reliable and the simulated data mimicked the real data correlation structure, the difference in power between the mixed‐effect models and the non‐parametric within‐period power was smaller than expected.

Most literature on the analysis of SWTs focuses on the use of mixed‐effect models.^14^ We assessed the cluster mixed‐effect model, and the cluster‐period mixed‐effect model because they are widely used in practice 7 and in methodological literature 18, 19 respectively. However, we found that neither of these models gave nominal coverage when our simulated data reflected the correlation structure seen in real data. Whilst this has been previously shown for the cluster mixed‐effect model,^8^, ^9^, ^10^ it is novel for the cluster‐period mixed‐effect model.

This is the first study to show that the magnitude of under‐coverage of CIs from the cluster mixed‐effects model when period effects vary between clusters is dependent on the ICC. This builds on previous research that noted this low coverage.^8^, ^9^, ^10^ Our simulation study also suggests that the mixed‐effect model may produce CIs with low coverage when used with only 9 clusters, similar to Barker et al.^26^ Use of a t‐distribution rather than the normal distribution could overcome this small sample issue.^27^ By contrast, the non‐parametric within‐period method gave correct coverage in all scenarios, including with as few as 9 clusters. This extends the work of Ji et al 9 and Wang and DeGrutolla 10 who also investigated the use of permutation tests for SWT analysis.

The parametric within‐period approach is not to be recommended for SWT analysis. It had low CI coverage in all scenarios and trial designs. The low CI coverage explains the small *P*‐value this method gave in our example. For this method, the number of parameters estimated is large relative to the number of clusters. For example, in the scenario with 11 sequences, the covariance matrix consists of 10 variance parameters and 45 covariance parameters for each pair of periods, 10 within‐period log odds‐ratios, and 10 control log odds, estimated with data from only 33 or 121 clusters.

The non‐parametric within‐period method had lower power than the mixed‐effects models when period effects were common to all clusters (ie, when the mixed‐effects model assumptions were true). In this scenario, the non‐parametric within‐period method had much higher variability in the estimated intervention effects, particularly when the ICC was high. This is consistent with previous findings.^4^, ^11^ However, when the period effect varied between clusters, the difference between the power of the non‐parametric within‐period method and the cluster‐period mixed‐effect model was reduced. This is despite the power of the cluster‐period mixed‐effect model being over‐estimated because of CI under‐coverage. This demonstrates that in realistic scenarios, the reduction in power from using a within‐period analysis is less than suggested by previous literature.^4^, ^11^ The main challenge is anticipating the correlation structure of the data when planning an analysis. Choosing a method that is robust irrespective of the correlation structure avoids pre‐specifying an analysis that is inappropriate.

We have found that the power of the non‐parametric within‐period method is dependent on the total number of clusters, and not on the number of sequences. This contradicts the design effects reported in Hayes and Moulton 2 where the power of a within‐period analysis decreased as the number of sequences increased. Hayes and Moulton explain that the loss of power is due to the increasing imbalance in the number of clusters in each condition within the periods. However, their calculations did not account for clustering or correlations between periods. In our analysis, the permutation tests account for all correlations in the data, and our findings suggest that this minimises the loss of power due to the imbalance in the number of clusters—perhaps even reversing it. Further research is needed on how the number of sequences affects the power of this methods in a wider range of scenarios. Consistent with previous research,^17^, ^28^ the statistical power of the mixed‐effects model was dependent on the number of sequences, as well as the number of clusters.

The within‐period methods required a heuristic correction for when the prevalence of the outcome in a cluster during a period was either 0 or 1 because of their use of a cluster‐level analysis. The heuristic was frequently required for trial designs with 11 sequences because each cluster‐period contained fewer observations in this design. Previous research has noted that this, and the use of cluster summaries to estimate an odds ratio, has led to a biased intervention effect estimate.^29^ In our simulation study, the intervention effect estimates calculated using cluster‐level methods were consistently slightly larger than the estimates from the mixed‐effect models, but they were within our pre‐specified boundary of 1/2 a standard deviation from the true effect, and the difference was not of practical relevance. The heuristic is not required for calculating risk difference or other difference estimates, such as mean differences or rate differences, but it would be required for ratio estimates.

Other analysis methods may exist or be developed that are able to provide robust estimates whilst including the between‐period comparisons. Research is needed into generalized estimating equations or the addition of robust standard errors in the context of SWTs. However, these methods still rely on having many clusters.^30^

A strength of the simulation study is that we used National Health Service health check uptake data at the local‐authority level to demonstrate the effect of realistic variability in period effects, leading to a realistic correlation structure. We explored the performance of the methods in a range of realistic settings by varying the ICC and looking at several different trial designs.

The simulation study has limitations. For our results to be most applicable to health research, we explored binary outcomes and so calculated odds ratios. Further research is required to examine whether the mixed‐effects models are more robust to misspecification with a continuous outcome, and to explore the properties of the non‐parametric within‐period method with continuous and rate outcomes. Other features of SWTs, such as delayed intervention, sparse data collection, or lags were not considered. The performance of the non‐parametric within‐period method requires further research in the presence of more extreme ICCs. A limitation of using cluster‐level analyses is that power is lost when there is high variability in cluster sizes. Whilst we allowed cluster size to vary, we did not explore the effect of different levels of variability on power. When there is high variability in cluster size, the method could be extended to weight clusters in the analysis within each period by their size, as described by Hayes and Moulton,^2^ to improve precision.

The weights used in the non‐parametric within‐period method were chosen to minimise the number of assumptions. The permutation test was chosen as this makes no assumptions about the composition of the data other than that of exchangeability and testing a strong null hypothesis (so assumes a common intervention effect).^31^ There may be improvements in power available from making assumptions about the data. However, adding assumptions may negate the benefits of the non‐parametric within‐period method, and Matthews and Forbes suggest that the gains will be small.^4^ Our inverse variance weights assumed the same variance in the intervention and control condition, but efficiency may be improved by relaxing this assumption in settings where the variance was expected to be different in each condition. The method could be extended to incorporate adjustment for baseline individual‐level covariates, as described by Hayes and Moulton.^2^

Presenting absolute as well as relative effect sizes can improve interpretation, as is recommended by the CONSORT statement.^32^ We have shown that the non‐parametric within‐period method can be simply adapted to provide estimates of risk differences. The method could also be extended to calculate risk ratios. Model‐based methods of calculating risk difference and risk ratios are available,^29^, ^33^ but further research is needed into their use in SWTs.

The non‐parametric within‐period approach to the analysis of SWTs, using permutation tests, is an unbiased and robust alternative to the commonly used mixed‐effects modelling approach. Researchers designing and analysing SWTs, in the common position of being uncertain about whether data will meet the assumptions necessary for mixed‐effect models, should consider this as an alternative method to emphasise that the SWT is a randomised trial.

## Supporting information

Data S1. A: R code for cluster summary analysis
B: Generating scenarios
C: Simulation study results use of heuristic adjustment for within‐period method
D1: Simulation study results—mean intervention effect log odds ratio estimates
D2: Simulation study results—variability of intervention effect log odds ratio estimates
E: Simulation study results—coverage
F: Simulation study results—power
