# Supplementary material for: Robust analysis of stepped wedge trials using cluster‐level summaries within periods
Source: Stat Med. 2018 Apr 10;37(16):2487–500. doi: 10.1002/sim.7668 (PMC6032886; doi:10.1002/sim.7668)
Supplement: Supplementary file 1 — Data S1. A: R code for cluster summary analysis B: Generating scenarios C: Simulation study results use of heuristic adjustment for within‐period method D1: Simulation study results—mean intervention effect log odds ratio estimates D2: Simulation study results—variability of intervention effect log odds ratio estimates E: Simulation study results—coverage F: Simulation study results—power [file SIM-37-2487-s001.docx]

# Supporting Information A: R code for cluster summary analysis

cluster.summary <- function(data, cluster, group, time, rx, summary, null) {

 f.effect <- function(x){

 #Aggregate data by time and intervention condition
 slices.long <- aggregate(summary ~ time + rx, data = x,
 FUN = function(X) c(mean = mean(X, na.rm = TRUE),
 n = sum(!is.na(X)),
 var = var(X, na.rm = TRUE)))

 slices.long$mean <- slices.long$summary[,1]
 slices.long$n <- slices.long$summary[,2]
 slices.long$var <- slices.long$summary[,3]

 #reshape so one row per time slice
 slices <- reshape(direction = "wide",
 data = slices.long[,c("time", "rx", "mean", "n", "var")],
 v.names = c("mean", "n", "var"),
 idvar = c("time"),
 timevar = "rx")

 #calculate difference
 slices$diff <- slices$mean.1 - slices$mean.0

 #Calculate a weight assuming the same variance in both arms

 slices$wgt <- ( (((slices$n.0 - 1) * slices$var.0 + (slices$n.1 - 1) * slices$var.1) /
 (slices$n.0 + slices$n.1 - 2)) * (1/slices$n.0 + 1/slices$n.1) )^-1

 weighted.mean(slices$diff, slices$wgt, na.rm = TRUE)
 }

 f.permute <- function(y, design) {

 design$cluster <- sample(design$cluster, replace = FALSE)

 design.long <- reshape(design,
 direction = "long",
 idvar = c("cluster", "group"))

 permuted.data <- merge(y[, c("cluster", "time", "summary")],
 design.long,
 by = c("cluster", "time"))

 return(f.effect(permuted.data))
 }

 dataset <- data.frame(cluster = eval(substitute(cluster), data),
 group = eval(substitute(group), data),
 time = eval(substitute(time), data),
 rx = as.numeric(eval(substitute(rx),data)),
 summary = eval(substitute(summary),data))


 dataset$summary <- dataset$summary - null * dataset$rx


 #Calculate observed effect
 mean.effect <- f.effect(dataset)

 #Create a dataset of the design
 design <- reshape(direction = "wide",
 idvar = c("cluster", "group"),
 timevar = "time",
 v.names = "rx",
 data = unique(dataset[,c("cluster","group","rx","time")]))

 #Calculate p value under null = 0
 permutations <- replicate(10000, f.permute(dataset, design), simplify = TRUE)

 p <- sum(abs(permutations) > abs(mean.effect)) / 10000


 return(c(mean.effect, p))

}

# Supporting Information B: Generating scenarios

We based the simulations on an outcome that might plausibly be the target of an SWT: uptake of NHS health checks, with plausible clusters: English local authorities.

Across England, GP surgeries and third parties offer all adults between the ages of 40-74 a health check to assess the patient's risk of diabetes, heart disease, kidney disease, stroke, and dementia [1]. A recent study found that while uptake of health checks has improved since their introduction uptake is still low; in 2012 30% of patients accepted the offer of a health check [2]. Data on the uptake of these health checks is published on the Public Health England website for each year quarter, by local authority. In this study we used data from 2013-2014 to simulate trials designed to study the effect of an unspecified intervention to improve uptake of health checks.

Local authorities were removed from the dataset if at any time point they recorded 100%, or 0% uptake of health checks, they had no offers of health checks, or they had more cases of health check uptake than offers. We then visually inspected the log odds of health check uptake to identify any outlying local authorities; a further 2 local authorities were removed as they had unusually high uptake in some quarters. This process removed a total of 29/152 local authorities.

In the remaining 123 local authorities we analysed the health check uptake to assess how uptake was changing over time, and how this varied between the local authorities. For each local authority we modelled the acceptance of health checks as:

$$logit\left( P\left( Y_{ijk}=1 \right) \right)=\mu_{i}+\beta_{1i}X_{1ij}+\beta_{2i}X_{2ij}^{\gamma_{i}}$$

Where $Y_{ijk}$ is the log odds of health check acceptance in local authority $i$ at time $j$, $\mu_{i}$ is the log odds of acceptance in local authority $i$ in the first quarter of 2013, $X_{1ij}$ is an indicator of year; 0 in 2013 and 1 in 2014, $\beta_{1i}$ is the log odds ratio comparing health check acceptance in 2014 to 2013 in local authority $i$, $X_{2ij}^{\gamma_{i}}$ is the quarter 1,2,3 or 4 within each year to a power $\gamma_{i}$ selected using fractional polynomials to allow for a non-linear trend (more details below), and $\beta_{2i}$ is the log odds ratio for quarter in local authority $i$. This meant that we were assuming the same effect of quarter in both years.

This model was run for each local authority using fractional polynomials to select a value $\gamma_{i}$ fo reach local authority. The most common value selected was $\gamma_{i}=3$.

We then ran a mixed effect model across all the local authorities where:

$$logit\left( P\left( Y_{ijk}=1 \right) \right)=\mu+u_{i}+(\beta_{1}+v_{i})X_{1ij}+(\beta_{2}+w_{i})X_{2ij}^{3}$$

$$\left( \begin{matrix} u_{i} \\ v_{i} \\ w_{i} \end{matrix} \right)\sim MultivariateNormal\left( \left( \begin{matrix} 0 \\ 0 \\ 0 \end{matrix} \right),\left( \begin{matrix} \sigma_{u} & cov(u_{i},v_{i}) & cov(u_{i},w_{i}) \\ cov(u_{i},v_{i}) & \sigma_{v} & cov(v_{i},w_{i}) \\ cov(u_{i},w_{i}) & cov(v_{i},w_{i}) & \sigma_{w} \end{matrix} \right) \right)$$

where $\mu$ is now the mean log odds of health check uptake in quarter 1 of 2013, $u_{i}$ is a random intecept, $v_{i}$ is a random effect for year, and $w_{i}$ is a random effect for quarter all with the multivariate normal distribution described above.

The resulting model gave coefficients $\mu=-0.14$, $\beta_{1}=-0.04$, and $\beta_{2}=0.01$ with the following multivariate normal distribution:

$$\left( \begin{matrix} u_{i} \\ v_{i} \\ w_{i} \end{matrix} \right)\sim MultivariateNormal\left( \left( \begin{matrix} 0 \\ 0 \\ 0 \end{matrix} \right),\left( \begin{matrix} 0.306 & -0.150 & -0.002 \\ -0.150 & 0.253 & -0.001 \\ -0.002 & -0.001 & 0.001 \end{matrix} \right) \right)$$

We simulated two scenarios for the period effects of quarter and year:

1. Period effect differs between clusters. The coefficients for quarter, year, and the constant for our clusters were sampled from the multivariate normal distribution as seen in the data and given above. This lead to the following correlation structure across the 10 time periods:

$$\left( \begin{matrix} 1.00 & 1.00 & 0.98 & 0.83 & 0.46 & 0.55 & 0.55 & 0.54 & 0.42 & 0.17 \\ 1.00 & 1.00 & 0.99 & 0.84 & 0.48 & 0.55 & 0.55 & 0.54 & 0.43 & 0.19 \\ 0.98 & 0.99 & 1.00 & 0.92 & 0.61 & 0.51 & 0.51 & 0.53 & 0.49 & 0.31 \\ 0.83 & 0.84 & 0.92 & 1.00 & 0.88 & 0.35 & 0.37 & 0.45 & 0.60 & 0.61 \\ 0.46 & 048 & 0.61 & 0.88 & 1.00 & 0.09 & 0.11 & 0.25 & 0.59 & 0.82 \\ 0.55 & 0.55 & 0.51 & 0.35 & 0.09 & 1.00 & 1.00 & 0.98 & 0.79 & 0.35 \\ 0.55 & 0.55 & 0.51 & 0.38 & 0.11 & 1.00 & 1.00 & 0.99 & 0.81 & 0.37 \\ 0.54 & 0.54 & 0.53 & 0.45 & 0.25 & 0.98 & 0.99 & 1.00 & 0.89 & 0.52 \\ 0.42 & 0.43 & 0.49 & 0.60 & 0.59 & 0.79 & 0.81 & 0.89 & 1.00 & 0.85 \\ 0.17 & 0.19 & 0.31 & 0.61 & 0.82 & 0.35 & 0.37 & 0.52 & 0.85 & 1.00 \end{matrix} \right)$$

1. Period effect common to all clusters. The mean coefficient for quarter and year were selected for all clusters and the constant was sampled from a normal distribution with mean and variance from the multivariate normal distribution. i.e.

$$\left( \begin{matrix} u_{i} \\ v_{i} \\ w_{i} \end{matrix} \right)\sim MultivariateNormal\left( \left( \begin{matrix} 0 \\ 0 \\ 0 \end{matrix} \right),\left( \begin{matrix} 0.306 & 0 & 0 \\ 0 & 0 & 0 \\ 0 & 0 & 0 \end{matrix} \right) \right)$$

This gave the data an exchangeable correlation structure.

We wanted to compare our analysis methods in data with a smaller ICC so we used two scenarios for the intra-cluster correlation (ICC).

1. High ICC: corresponding to the original data and the original multivariate normal distribution. When period effects differed between clusters this meant that the ICC varied between 0.06 and 0.15 over the two years. When period effects were common to all clusters this meant that the ICC remained the same as the ICC in the first quarter of 2013 in the original data, so ICC=0.08 throughout.
2. Low ICC: The between cluster variance was reduced by multiplying the covariance matrix by 0.2. When period effects differed between the clusters this meant that the ICC varied between 0.01 and 0.03 over the two years. When period effects were common to all cluster this meant that the ICC remained at ICC=0.02 throughout.

This gave us four data scenarios for how acceptance of health checks varied over time, and how this varied between local authorities.

# Supporting Information C: Simulation study results use of heuristic adjustment for within-period method

| Common or varying period effects | ICC | Steps | Clusters per group | Number of simulations where heuristic was used | Percentage of simulations where heuristic was used | Mean number of times used per dataset |
| --- | --- | --- | --- | --- | --- | --- |
| Fixed | 0.02 | 3 | 3 | 0 | 0 | 0.0 |
| Fixed | 0.02 | 3 | 11 | 0 | 0 | 0.0 |
| Fixed | 0.02 | 11 | 3 | 97 | 10 | 0.1 |
| Fixed | 0.02 | 11 | 11 | 842 | 84 | 1.8 |
| Fixed | 0.08 | 3 | 3 | 0 | 0 | 0.0 |
| Fixed | 0.08 | 3 | 11 | 1 | 0 | 0.0 |
| Fixed | 0.08 | 11 | 3 | 422 | 42 | 0.6 |
| Fixed | 0.08 | 11 | 11 | 990 | 99 | 5.1 |
| Varying | 0.02 | 3 | 3 | 0 | 0 | 0.0 |
| Varying | 0.02 | 3 | 11 | 0 | 0 | 0.0 |
| Varying | 0.02 | 11 | 3 | 117 | 12 | 0.1 |
| Varying | 0.02 | 11 | 11 | 884 | 88 | 2.0 |
| Varying | 0.08 | 3 | 3 | 0 | 0 | 0.0 |
| Varying | 0.08 | 3 | 11 | 0 | 0 | 0.0 |
| Varying | 0.08 | 11 | 3 | 638 | 64 | 1.0 |
| Varying | 0.08 | 11 | 11 | 1000 | 100 | 7.1 |

# Supporting Information D1: Simulation study results- Mean intervention effect log odds ratio estimates

| Common or Varying period effects | ICC | Number of sequences | Clusters per Sequence | Non-parametric within-period method | Parametric within-period method | Cluster model | Cluster-period model |
| --- | --- | --- | --- | --- | --- | --- | --- |
| Common | 0.02 | 3 | 3 | 0.27 | 0.27 | 0.26 | 0.27 |
| Common | 0.02 | 3 | 11 | 0.26 | 0.26 | 0.26 | 0.26 |
| Common | 0.02 | 11 | 3 | 0.28 | 0.28 | 0.26 | 0.26 |
| Common | 0.02 | 11 | 11 | 0.28 | 0.28 | 0.26 | 0.26 |
| Common | 0.08 | 3 | 3 | 0.28 | 0.27 | 0.26 | 0.26 |
| Common | 0.08 | 3 | 11 | 0.28 | 0.28 | 0.26 | 0.26 |
| Common | 0.08 | 11 | 3 | 0.28 | 0.28 | 0.26 | 0.26 |
| Common | 0.08 | 11 | 11 | 0.28 | 0.28 | 0.27 | 0.27 |
| Varying | 0.02 | 3 | 3 | 0.24 | 0.24 | 0.24 | 0.24 |
| Varying | 0.02 | 3 | 11 | 0.26 | 0.26 | 0.26 | 0.26 |
| Varying | 0.02 | 11 | 3 | 0.26 | 0.27 | 0.26 | 0.26 |
| Varying | 0.02 | 11 | 11 | 0.28 | 0.28 | 0.26 | 0.26 |
| Varying | 0.08 | 3 | 3 | 0.24 | 0.24 | 0.22 | 0.22 |
| Varying | 0.08 | 3 | 11 | 0.24 | 0.24 | 0.24 | 0.24 |
| Varying | 0.08 | 11 | 3 | 0.27 | 0.27 | 0.25 | 0.26 |
| Varying | 0.08 | 11 | 11 | 0.27 | 0.27 | 0.25 | 0.26 |

# Supporting Information D2: Simulation study results- Variability of intervention effect log odds ratio estimates

| Common or Varying period effects | ICC | Number of sequences | Clusters per Sequence | Non-parametric within-period method | Parametric within-period method | Cluster model | Cluster-period model | R Parametric within-period method | R Cluster model | R Cluster-period model |
| --- | --- | --- | --- | --- | --- | --- | --- | --- | --- | --- |
| Common | 0.02 | 3 | 3 | 0.19 | 0.20 | 0.15 | 0.15 | 0.96 | 1.70 | 1.60 |
| Common | 0.02 | 3 | 11 | 0.10 | 0.10 | 0.08 | 0.08 | 1.00 | 1.59 | 1.56 |
| Common | 0.02 | 11 | 3 | 0.10 | 0.11 | 0.07 | 0.07 | 0.91 | 1.99 | 1.98 |
| Common | 0.02 | 11 | 11 | 0.05 | 0.05 | 0.04 | 0.04 | 0.99 | 2.08 | 2.08 |
| Common | 0.08 | 3 | 3 | 0.38 | 0.40 | 0.18 | 0.18 | 0.91 | 4.58 | 4.35 |
| Common | 0.08 | 3 | 11 | 0.20 | 0.20 | 0.10 | 0.11 | 0.97 | 3.46 | 3.44 |
| Common | 0.08 | 11 | 3 | 0.19 | 0.19 | 0.08 | 0.08 | 0.97 | 6.01 | 6.00 |
| Common | 0.08 | 11 | 11 | 0.10 | 0.10 | 0.04 | 0.04 | 1.00 | 5.86 | 5.85 |
| Varying | 0.02 | 3 | 3 | 0.17 | 0.18 | 0.17 | 0.16 | 0.89 | 1.05 | 1.12 |
| Varying | 0.02 | 3 | 11 | 0.09 | 0.09 | 0.09 | 0.09 | 0.99 | 1.02 | 1.08 |
| Varying | 0.02 | 11 | 3 | 0.09 | 0.10 | 0.08 | 0.08 | 0.87 | 1.42 | 1.45 |
| Varying | 0.02 | 11 | 11 | 0.05 | 0.05 | 0.04 | 0.04 | 0.98 | 1.52 | 1.53 |
| Varying | 0.08 | 3 | 3 | 0.34 | 0.36 | 0.37 | 0.33 | 0.89 | 0.88 | 1.10 |
| Varying | 0.08 | 3 | 11 | 0.17 | 0.17 | 0.18 | 0.15 | 0.98 | 0.90 | 1.19 |
| Varying | 0.08 | 11 | 3 | 0.17 | 0.18 | 0.13 | 0.12 | 0.90 | 1.91 | 2.01 |
| Varying | 0.08 | 11 | 11 | 0.09 | 0.09 | 0.07 | 0.06 | 1.00 | 1.75 | 1.89 |

# Supporting Information E: Simulation study results- Coverage

| Common or Varying period effects | ICC | Number of sequences | Clusters per Group | Non-parametric within-period method | Parametric within-period method | Cluster model | Cluster-period model |
| --- | --- | --- | --- | --- | --- | --- | --- |
| Common | 0.02 | 3 | 3 | 94.3 | 78.0 | 92.0 | 92.0 |
| Common | 0.02 | 3 | 11 | 95.7 | 92.6 | 94.6 | 94.9 |
| Common | 0.02 | 11 | 3 | 94.4 | 71.9 | 94.1 | 94.4 |
| Common | 0.02 | 11 | 11 | 93.6 | 78.8 | 95.6 | 95.6 |
| Common | 0.08 | 3 | 3 | 94.9 | 76.5 | 94.1 | 94.6 |
| Common | 0.08 | 3 | 11 | 94.3 | 89.4 | 93.8 | 94.4 |
| Common | 0.08 | 11 | 3 | 95.9 | 60.1 | 94.5 | 94.5 |
| Common | 0.08 | 11 | 11 | 93.5 | 66.3 | 94.5 | 94.7 |
| Varying | 0.02 | 3 | 3 | 96.0 | 75.8 | 86.6 | 90.1 |
| Varying | 0.02 | 3 | 11 | 95.4 | 91.7 | 90.6 | 94.4 |
| Varying | 0.02 | 11 | 3 | 95.0 | 76.5 | 90.6 | 93.0 |
| Varying | 0.02 | 11 | 11 | 93.7 | 84.7 | 92.9 | 94.9 |
| Varying | 0.08 | 3 | 3 | 95.3 | 75.4 | 59.5 | 85.2 |
| Varying | 0.08 | 3 | 11 | 95.4 | 91.4 | 71.6 | 92.9 |
| Varying | 0.08 | 11 | 3 | 94.5 | 63.1 | 76.2 | 89.9 |
| Varying | 0.08 | 11 | 11 | 96.3 | 76.2 | 76.8 | 91.5 |

# Supporting Information F: Simulation study results- Power

| Common or varying period effects | ICC | Number of sequences | Clusters per Group | Non-parametric within-period method | Parametric within-period method | Cluster model | Cluster-period model |
| --- | --- | --- | --- | --- | --- | --- | --- |
| Common | 0.02 | 3 | 3 | 27 | 52 | 49 | 47 |
| Common | 0.02 | 3 | 11 | 73 | 77 | 92 | 90 |
| Common | 0.02 | 11 | 3 | 78 | 94 | 96 | 96 |
| Common | 0.02 | 11 | 11 | 100 | 100 | 100 | 100 |
| Common | 0.08 | 3 | 3 | 11 | 33 | 36 | 34 |
| Common | 0.08 | 3 | 11 | 30 | 35 | 73 | 70 |
| Common | 0.08 | 11 | 3 | 32 | 74 | 93 | 93 |
| Common | 0.08 | 11 | 11 | 80 | 95 | 100 | 100 |
| Varying | 0.02 | 3 | 3 | 22 | 49 | 45 | 40 |
| Varying | 0.02 | 3 | 11 | 80 | 85 | 89 | 85 |
| Varying | 0.02 | 11 | 3 | 80 | 93 | 95 | 94 |
| Varying | 0.02 | 11 | 11 | 100 | 100 | 100 | 100 |
| Varying | 0.08 | 3 | 3 | 10 | 32 | 45 | 22 |
| Varying | 0.08 | 3 | 11 | 28 | 36 | 63 | 36 |
| Varying | 0.08 | 11 | 3 | 36 | 73 | 80 | 68 |
| Varying | 0.08 | 11 | 11 | 87 | 98 | 100 | 99 |

# References

1. NHS. Health check data 2015.

2. Robson J, Dostal I, Sheikh A, Eldridge S, Madurasinghe V, Griffiths C *et al.* The nhs health check in england: An evaluation of the first 4 years. *BMJ Open* 2016; **6**(1): e008840. DOI: [10.1136/bmjopen-2015-008840](https://doi.org/10.1136/bmjopen-2015-008840).
